# Antibiotic Stewardship in Patients With Acute Bronchitis: A Case Report of Doxycycline-Induced Esophagitis

**DOI:** 10.7759/cureus.26354

**Published:** 2022-06-26

**Authors:** Muhammad Ali Butt, Mark Peicher, Anthony P Nguyen, Abu Baker Sheikh

**Affiliations:** 1 Internal Medicine, Allegheny Health Network, Pittsburgh, USA; 2 Department of Internal Medicine, University of New Mexico Health Sciences Center, Albuquerque, USA; 3 Internal Medicine, University of New Mexico School of Medicine, Albuquerque, USA

**Keywords:** excessive use of antibiotics, acute bronchitis, esophagitis, doxycycline, antibiotic stewardship

## Abstract

Acute bronchitis is a self-limiting disease, characterized by mild constitutional symptoms and a cough lasting two to three weeks. The disease usually occurs secondary to viruses; therefore, only symptomatic and supportive care is advised for the patients. Despite the recommended guidelines, most patients are prescribed antibiotics. Here, we present a case of a 38-year-old female who presented to the hospital with a sudden onset of severe epigastric pain. The patient recently started a 10-day course of doxycycline for acute bronchitis. She was admitted, evaluated, and diagnosed with doxycycline-induced esophagitis, and managed accordingly. This report highlights how excessive use of antibiotics is leading to adverse effects, antibiotic resistance, increased health care costs, and invasive testing. It also emphasizes the importance of antibiotic stewardship.

## Introduction

Acute bronchitis is defined as an acute inflammation of the large airways without any signs of pneumonia. It is characterized by mild constitutional symptoms and cough (productive or non-productive), which typically lasts for two to three weeks. The disease can be caused by viruses (most common), allergens, and irritants. Acute bronchitis is a self-limiting disease; therefore, only supportive and symptomatic treatment is recommended [1].

In contrast to the recommended guidelines for uncomplicated acute bronchitis, many of these patients are still prescribed antibiotics. This excessive and unnecessary use of antibiotics has resulted in increased antibiotic-associated adverse effects, antibiotic resistance, and health care costs [2]. Herein, we report a case of a 38-year-old female who was treated with doxycycline for acute bronchitis and later presented to the emergency department with symptoms of esophagitis, which occurred secondary to doxycycline use. This case highlights the unnecessary use of antibiotics for acute bronchitis and the associated adverse effects. It also highlights the increased antibiotic resistance and burden on health care caused by prescribing excessive antibiotics and the strategies that should be implemented to reduce the rate of antibiotic prescription.

## Case presentation

A 38-year-old female with no past medical history presented to the emergency department with epigastric pain for two days. The pain was described as a squeezing, sharp pain that radiated to her back. According to the patient, the pain began suddenly with no inciting factors. Twenty-four hours later, her symptoms progressed and the pain intensity worsened to a 10/10. She denied any fever, chills, melena, or hematemesis. Her last menstrual period was a few days prior to presentation, and it was regular. Of note, she was recently given a 10-day course of Doxycycline, and her last dose was the night prior to the onset of her symptoms. She denied any recent travel, dietary changes, work-related exposures, or over-the-counter non-steroidal anti-inflammatory drug (NSAID) use.

On presentation, the patient was afebrile. She had a respiratory rate of 24 breaths per minute, a normal heart rate, and a blood pressure of 101/63 mm Hg. Her physical exam was remarkable for epigastric tenderness to palpation. Her complete blood count was remarkable for a white blood cell count of 12.7 and a hemoglobin of 10.7 mg/dL. A complete metabolic panel, lactate, and lipase were unremarkable. Troponin and beta-human chorionic gonadotropin were negative. An electrocardiogram demonstrated normal sinus rhythm. A chest X-ray was negative for any findings. The abdominal X-ray demonstrated a non-obstructive bowel gas pattern. The CT abdomen/pelvis was negative for any pathology.

**Figure 1:**
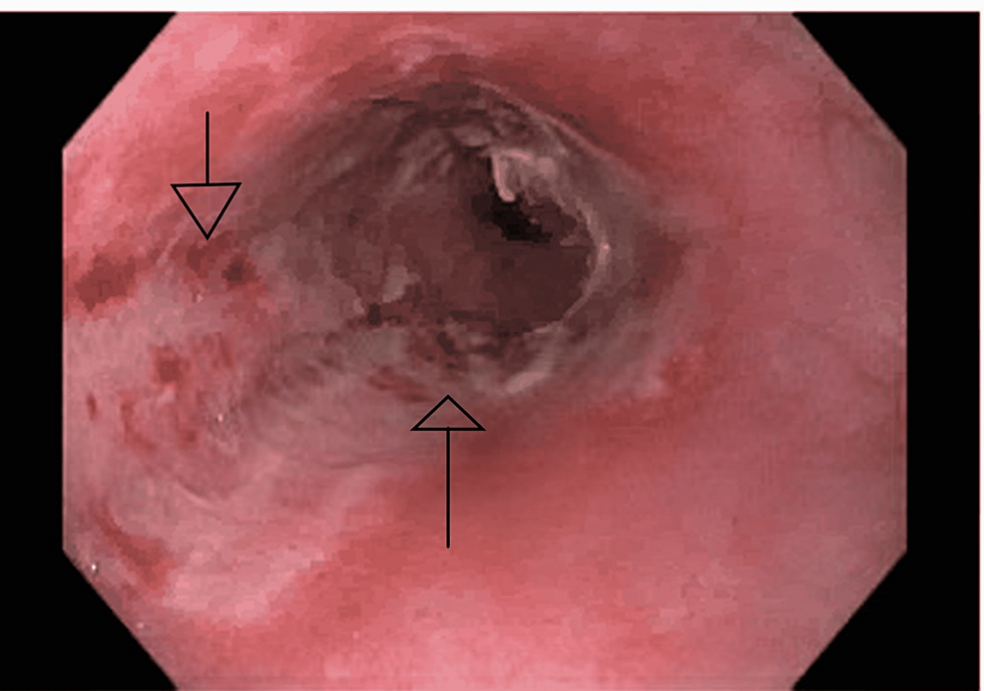
LA grade D esophagitis.

**Figure 2:**
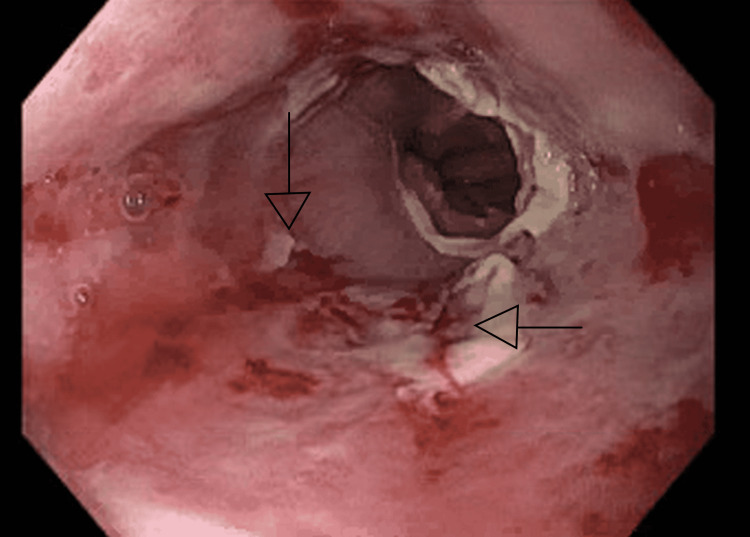
LA grade D esophagitis with bleeding.

She was started on pantoprazole 40 mg IV twice daily, paracetamol and morphine for pain, and ondansetron for nausea. The patient underwent esophagogastroduodenoscopy (EGD) and was found to have LA grade D esophagitis with oozing as shown in Figures 1-2. All other exam findings on EGD were normal. She was recommended to continue omeprazole 40 mg orally twice daily for 12 weeks, and then repeat upper endoscopy to evaluate the response to therapy. She was discharged home in a stable condition. Ultimately, the patient was diagnosed with pill-induced esophagitis due to doxycycline, which she was given to treat bronchitis.

## Discussion

Acute bronchitis is among the top ten most common illnesses among outpatient clinics in the United States [3]. It is a self-limiting disease; therefore, according to guidelines, antibiotics are not recommended for its treatment [1]. Despite this, acute bronchitis is the most common disease for which antibiotics are prescribed in outpatient settings around the world [4].

This excessive use of antibiotics has resulted in more adverse effects, such as allergic reactions, nausea, vomiting, and *Clostridium difficile* infection [1]. Our case also reports an adverse effect: doxycycline-induced esophagitis, which further highlights the fact that inappropriate use of antibiotics is not only leading to adverse effects but also to increased patient exposure to invasive testing, which can otherwise be easily prevented. According to an estimate, the incidence of drug-induced esophagitis is 3.9 per 100,000 people per year, and doxycycline is the most common and under-reported cause of pill esophagitis [5].

Apart from the adverse effects, inappropriate antibiotic use has led to increased antibiotic resistance, which has resulted in an epidemic of antibiotic-resistant infections, further resulting in increased morbidity, mortality, and economic costs due to infections [2]. The deaths caused by resistant infections are estimated to exceed 10 million per year by 2050 [6].

Antibiotic stewardship is an important means of limiting antibiotic resistance. The first time the term "stewardship" was used in reference to antibiotics was in 1996, in an article in New Horizons [7]. The main goal of antibiotic stewardship is to provide better patient care by not only limiting the excess use of antibiotics but also focusing on appropriate drug selection, dosing, and duration of use, in order to achieve maximum results [8].

The core elements of a successful antibiotic stewardship program are: strong leadership, tracking of antibiotic use, regular reporting of antibiotic use and resistance, educating providers on use and resistance, and specific interventions [9]. The specific interventions which have provided significant results include prospective audit and feedback, antibiotic time-outs, and disease/syndrome specific approaches, for example, targeting prescriptions for acute respiratory tract infections [10].

Moreover, according to a study done by Fishmann, patients in the antibiotic stewardship group were three times more likely to get appropriate treatment as per guidelines; they were twice as likely to be cured and they were 80% less likely to have treatment failure, as compared to the patients receiving standard care [8].

Hence, antibiotic stewardship, which has evolved immensely over the past 20 years and now has become a global drive, plays a key role in optimizing antibiotic use. However, there are still some hindrances to its success, such as not enough trained personnel, limited structural and time resources, and variable cultural determinants [7].

Therefore, in order to achieve a long-lasting change in prescribing behaviors and a successful reduction in unnecessary use of antibiotics, a global standard for the successful implementation of these programs should be set, and physicians and patients should realize the importance of antibiotic stewardship and take every step to achieve this goal for a better future.

## Conclusions

Acute bronchitis is a self-limiting disease and does not warrant the need for antibiotics. Despite this, antibiotics such as doxycycline are commonly prescribed for it in outpatient settings, leading to multiple adverse effects, i.e., pill esophagitis as seen in our case, requiring invasive intervention. Inappropriate antibiotic prescribing practices become a seed for antibiotic resistance and the spread of antibiotic-resistant infections, leading to increased morbidity, mortality, invasive testing, length of stay, health care expenditure, and poor outcomes. Antibiotic stewardship programs play a pivotal role in medicine, by improving patient outcomes, combating antibiotic resistance, and reducing the overall health cost. Therefore, strategies should be made for the successful implementation of the programs globally and for achieving significant results.
